# Nutrition, Body Composition, and Blood Pressure in Children and Adolescents from the Korea National Health and Nutrition Examination Survey

**DOI:** 10.3390/ijerph192013272

**Published:** 2022-10-14

**Authors:** Susan Taejung Kim, Young-Hwan Song

**Affiliations:** Department of Pediatrics, Seoul National University Bundang Hospital, Seongnam-si 13620, Gyeonggi-do, Korea

**Keywords:** blood pressure, pediatrics, body composition, nutrition, diet

## Abstract

We aimed to investigate the association between nutrition and blood pressure and the role that body composition plays in this relationship. Korea National Health and Nutrition Examination Survey data from the years 2008–2020 were reviewed. A total of 11,234 subjects (5974 boys and 5260 girls) aged 10–18 years of age were selected. We analyzed the correlation between nutrition (intakes of energy, protein, fat, carbohydrate, sodium, saturated fatty acid (SFA), unsaturated fatty acid (USFA), and dietary fiber (DF)) and body composition (height, weight, waist circumference (WC), body mass index (BMI), and waist to height ratio (WHtR)), and performed multiple regression analysis to find the independent correlation between body composition and blood pressure (BP). We then compared the correlation between nutrition and BP, with or without adjustment for body composition. The intakes of energy, protein, fat, carbohydrate, sodium, and USFA had positive associations with height, weight, WC, and BMI. Systolic BP (SBP) and diastolic BP (DBP) were independently positively correlated with height and BMI. The intakes of energy, protein, fat, carbohydrate, sodium, and SFA had positive correlations with SBP and DBP, which disappeared when additionally adjusted for BMI and height. In conclusion, nutrition seems to affect BP via height and BMI in Korean children and adolescents.

## 1. Introduction

Hypertension, a major public health problem, increases the risk of cerebral and cardiovascular complications such as stroke and coronary vascular disease [1]. Cardiovascular complications account for from one third to one fourth of the total number of deaths in developed countries and are the leading causes of morbidity and mortality [2]. It is now evident that higher blood pressure (BP) in childhood may lead to higher BP in adulthood, an earlier onset of hypertension, and an increased risk of cardiovascular events [3,4,5]. High BP-related cardiovascular damage in youth has been demonstrated in both imaging and autopsy-based studies [6,7]. Thus, the identification of risk factors associated with hypertension in the pediatric populations is important.

Physical activity, family history, and smoking are some of the suggested contributing factors for hypertension. According to previous studies, dietary factors also affect BP. Specifically, a high consumption of fruit, vegetable and low-fat dairy may reduce BP, while an excessive consumption of saturated fat, red meat and sugar may have the opposite effect [8,9,10]. Other studies have also shown that an increase in sodium intake leads to a higher BP [11].

Body composition, such as obesity, is another well-known risk factor for hypertension [12], and obese children are more likely to develop hypertension than children with normal weight. One study estimated the increase to be three- or four-fold [13,14]. Known causes of obesity include genetic conditions, mood and the excess intake of food energy compared to its use [15]. Total energy intake has been increasing in children with the increasing availability of sugar-sweetened beverages, snacks, and fast food that is high in saturated fat. Such dietary factors is an important contributing factor to childhood obesity [16,17].

Many studies based on data from the Korea National Health and Nutrition Examination Survey (KNHANES) have attempted to determine the relationship between hypertension and body composition, between diet and body composition and between diet and hypertension in Korean children and adolescents. In Korean children and adolescents aged from 10 to 18 years, systolic blood pressure (SBP) was elevated by 3.9 mmHg from 2007–2009 to 2013–2015. Influencing factors were male sex, increasing age, and higher BMI z-scores or height z-scores [18]. A high waist circumference (WC) also showed a positive relationship with the incidence of cardiovascular risk factors in adolescents [19]. From the years 2007 to 2017, the prevalence of obesity increased from 8.7% to 15.0% in children aged from 6 to 18 years [20], while fat intake increased significantly from 20.3% of energy to 23.3% during the same period [21]. The percentage of protein intake also increased in children aged from 10 to 18 from 14.1% to 14.6%, when compared between periods of 2007–2009 and 2016–2018 [22]. Total calorie, carbohydrate, protein and salt intakes were also shown to have a significant positive association with SBP and obesity in this group [22]. In another study, dietary and urinary sodium showed a positive correlation with adiposity even after adjusting for the consumption of sugar-sweetened beverages in those aged between 10 to 18 years [23].

Previous studies based on KNHANES data have shown that nutrition intake and obesity affect each other as well as BP [24]. However, to our knowledge, no study based on KNHANES has examined the association between blood pressure and nutritional factors, as well as body composition, especially in the pediatric population. Thus, in this study, we aimed to investigate the relationship between nutritional intake and blood pressure, and, if any, the role that body composition plays in this relationship in Korean children and adolescents using the KNHANES data.

## 2. Materials and Methods

### 2.1. Study Population

KNHANES is a nationwide survey conducted annually by the Korea Centers for Disease Control and Prevention and the Ministry of Health and Welfare. The survey collected health and nutrition-related data from stratified multistage probability samples of Korean households. The details of the KNHANES survey have been documented elsewhere [25]. Subjects from the years 2008–2020 and ages 10–18 with recorded anthropometric data and blood pressure measurements were included in the present study. Subjects with renal failure or congenital heart disease were excluded. Data extraction was performed by both co-authors separately.

The KNHANES protocol was approved by the Institutional Review Board of Seoul National University Bundang Hospital (IRB No. X-1604-344-901).

### 2.2. Anthropometric Measurements

In KNHANES, anthropometric data, including height, weight and WC, were obtained by well-trained medical staffs using calibrated equipment. Measurements were performed according to standardized protocols. Height and WC were measured to the nearest 0.1 cm, and weight to the nearest 0.1 kg, using a stadiometer (Seca 225, Seca, Hamburg, Germany) for height, nonelastic tape for WC and an electronic balance (GL-6000-20, G-tech, Birmingham, MI, USA) for weight. Body mass index (BMI) was calculated as body weight in kilograms divided by the square of height in meters (kg/m^2^), and weight-to-height ratio (WHtR) by dividing weight in kilograms by height in centimeters. Reference data from the Korea Disease Control and Prevention Agency (https://knhanes.kdca.go.kr, accessed on 24 September 2022) were used to determine age- and sex-adjusted percentile for BMI, and a cut-off value of the 95th percentile was used to define high BMI [26]. The cut off value for high WHtR was 0.5 [27].

### 2.3. Blood Pressure Measurements

In KNHANES, BP was measured using a mercury sphygmomanometer with a properly sized cuff at a sitting position after 5 min of rest. Three measurements were taken at 30-s intervals from the right arm positioned at the level of the heart. The averages of the second and third measurements were used for the analysis. Intraclass correlation coefficients for SBP and diastolic blood pressure (DBP) between the second and third measurements were 0.956 (95% CI: 0.952–0.959) and 0.949 (95% CI: 0.952–0.959), respectively. Normative data published in the Korean Circulation Journal in 2009 were used to determine age, sex, and height-adjusted percentiles for BP [28]. Hypertension was defined as SBP and/or DBP over the 95th percentile for children aged <16 years, and SBP over 140 mmHg and/or DBP over 90 mmHg for those over 16 years old, as defined in the European Society of Hypertension guidelines published in 2016 [29]. Subjects taking antihypertensive medication were also grouped into the hypertension group, regardless of BP.

### 2.4. Nutritional Intake Estimation

In KNHANES, two dietitians visited households and conducted face-to-face interviews for a nutrition survey. They used a 24-h dietary recall method to gather information about the food the participant consumed the previous day, and a conversion process was carried out using the nutrition database from the Korea Health Industry Development Institute of the Ministry of Health and Welfare, Korean National Institute of Agricultural Sciences, Korean National Institute of Fisheries Sciences, Korean Ministry of Food and Drug Safety, Japanese Ministry of Education, Culture, Sports, Science and Technology, and the United States Department of Agriculture for the evaluation of energy and nutrient intakes using the following formula: energy and nutrient intake amount = intake frequency × food intake amount × energy and nutrient content by food item. The total energy, protein, fat, carbohydrate, saturated fatty acid (SFA), unsaturated fatty acid (USFA), dietary fiber (DF), and sodium intake were used in the analysis.

### 2.5. Statistical Analysis

SPSS version 23 (SPSS Inc., Chicago, IL, USA) was used for the statistical analysis. The anthropometric data, blood pressure, and nutritional intakes of the selected subjects were retrieved from the KNHANES database. An analysis of covariance was used to compare age, body composition (height, weight, WC, BMI, WHR), BP and nutrition intakes (energy, protein, fat, carbohydrate, sodium, SFA, USFA, DF) between boys and girls and between hypertensive and non-hypertensive groups. We estimated the multivariable-adjusted prevalence of hypertension, high BMI and high WHR using logistic regression analysis using R statistical programming language. Correlation analysis was used to deduce the relationship between body composition and nutrition intake. Multiple regression analysis was used to analyze the independent effects of body composition on BP. We performed a multivariable adjusted correlation analysis to evaluate the associations between nutrition intake and BP and the effects of body composition on these associations. Subjects taking antihypertensive medication were excluded in the analysis relating to BP. As the KNHANES followed a multi-stage clustered probability design for the sampling plan, we applied sample weights for the analysis. Statistical analyses were adjusted for age and/or sex. Two-tailed *p*-values less than 0.05 were considered statistically significant.

## 3. Results

Table 1 shows the characteristics of the study population. In total, 11,234 subjects (5974 boys and 5260 girls) were included in the analysis. In the comparison between boys and girls, body composition (height, weight, WC, BMI, WHtR) and nutritional intake (energy, protein, fat, carbohydrate, sodium, SFA, USFA, DF) were significantly higher in boys than girls (*p*-values < 0.001). The prevalence of hypertension and high WHtR were higher in boys than girls (*p*-values < 0.001), while differences in the prevalence of a high BMI were insignificant (*p*-value = 0.189).

In the analysis of the correlation between body composition and nutrition intake, height and weight showed positive correlations with the amount of energy, protein, fat, carbohydrate, sodium, SFA and USFA intakes, but showed insignificant correlations with DF. WC and BMI showed positive correlations with the intakes of energy, protein, fat, carbohydrate, sodium and SFA, but insignificant correlations with USFA and DF. WHtR, on the other hand, only showed a significant correlation with sodium intake (Table 2).

In multiple regression analyses showing independent correlations between variables, both SBP and DBP showed positive associations with height and BMI, but not with WHtR (Table 3).

In the correlation analysis between BP and nutrition intake with adjustments for sex and age only, SBP and DBP showed positive correlations with the intakes of energy, protein, fat, carbohydrate, sodium and SFA, but not with the intakes of USFA or DF. As height and BMI were shown to be independently associated with BP in Table 3, we investigated the role of height and BMI in this association between nutrition intake and BP. With additional adjustment for height or BMI, the positive associations between nutrition intake and BP still mostly remained, but were considerably attenuated. When additional adjustments were made for both height and BMI, these positive associations disappeared (Table 4).

Lastly, when comparing between hypertensive and normotensive subjects, no significant differences according to age and height existed between the two groups. Weight, WC, BMI, WHtR, SBP, DBP and the prevalence of high BMI and high WHtR were significantly higher in the hypertensive group. Energy, protein, fat, carbohydrate, sodium, and SFA intakes were also significantly higher in hypertensive subjects. There were no significant differences in USFA and DF intakes in these two groups (Table 5).

## 4. Discussion

In the present study on Korean children and adolescents, there were positive correlations between BP and the intakes of energy, protein, fat, carbohydrate, sodium and SFA, in accordance with previous studies. However, the relationship disappeared when adjusted for height and BMI. Therefore, it seems that nutrition intake affects BP via its effect on height and BMI.

The relationship between BP and diet has been recognized in previous studies and the effect of dietary approach on lowering BP was first seen more than 20 years ago [30]. Decreased blood pressure with reduced salt intake has been demonstrated in adults [31], as well as in pediatric groups [32]. Moreover, although results are equivocal on the effect of potassium on blood pressure in the pediatric group, a long term study (average follow-up of 7 years) on 233 Dutch children aged 5–17 years at baseline showed that a higher potassium intake might be related to lower BP [33]. Dietary guidelines to decrease the risk of hypertension and cardiovascular comorbidities incorporated such findings and focus on reducing the intake of sodium, sweetened beverages and processed carbohydrates, and increasing the intake of fruit and vegetables that are high in potassium. One such dietary plan is the Dietary Approaches to Reduce Hypertension, or DASH diet plan. The DASH diet is rich in fruits, vegetables, and low-fat dairy and low in saturated fat, red meat, and sweets [10]. In a recent meta-analysis by Filippou et al., 30 randomized clinical trials on adults were analyzed, and it was shown that the DASH diet significantly reduces SBP and DBP regardless of the baseline BP, and BP decreases were achieved in both hypertensive and non-hypertensive subjects [34]. The Mediterranean diet, another example of a dietary plan to tackle hypertension, is rich in whole grains, vegetables, fruits, nuts and extra-virgin olive oil, and was also shown to have a favorable effect on BP control [35]. A high intake of fruits, vegetables, and low-fat dairy products was also shown to lower BP in childhood [36,37,38,39].

Dietary plans for BP reduction also had positive effects on body composition. A meta-analysis in 2016 showed that the DASH diet significantly reduced body weight, BMI and WC [40]. Similarly, a study based on Spanish university students (N = 244) showed lower visceral fat and WC in addition to lower BP, in students who adhered more strictly to the DASH dietary pattern [41] and a systematic review of the 2018 literature showed the beneficial effect of the DASH diet on BP as well as obesity in adolescents [42]. A weight reduction of approximately 5 kg led to a BP reduction of 3.6–4.4 mmHg in a meta-analysis of 25 randomized controlled studies on adult subjects [43], and a decrease in the degree of obesity showed decreased BP in the pediatric groups as well [44].

A significant positive relationship between BMI and BP was seen in a study based on 101,606 Unites States children and adolescents aged from 3 to 17 years, and the risk of hypertension was raised by more than threefold in those who became obese or remained obese compared to those who were not obese [45]. A suggested pathophysiology explaining how obesity causes BP elevation is the activation of the sympathetic nervous system by the dysfunctional activation of the neurohormonal aspect of the adipocytes that are increased in obese people. Increased sympathetic nervous system activation not only directly causes vasoconstriction of the renal vascular bed but also leads to an increase in renin-angiotensin-aldosterone system activity.

Obesity-related dyslipidemia may also contribute to the elevation of BP [46]. In a study by Holms et al., a significant decrease in BP was shown in obese children and adolescents with weight reduction intervention. In this study, as some of the subjects regained weight after the termination of the weight reduction intervention, BP increased during the weight-regaining phase [47].

The above studies show that both nutritional factors and body composition influence BP, and that nutrition and body composition are also highly correlated. However, to the best of our knowledge, no studies have investigated the inter-relationship between these three components, especially in the pediatric group. Thus, this study is valuable in that we propose that nutrition influences BP through its effect on body composition in children and adolescents.

This study is limited by its retrospective, cross-sectional nature, making it difficult to deduce long-term effects or confirm any causal effect. Further long-term studies on the effect of nutritional intake on blood pressure would allow for better planning for the prevention of hypertension, beginning at pediatric ages.

## 5. Conclusions

The aim of preventing and treating hypertension in the pediatric group is to reduce the risk of hypertension and related cardiovascular diseases in adulthood. The treatment strategy includes lifestyle modifications, such as a healthy diet and adequate physical activity, and pharmacologic therapies. We believe that this study’s conclusion that diet might influence BP via its effect on body composition could allow for a better understanding of the pathophysiology of hypertension in the pediatric group, leading to better strategies to prevent hypertension in this group.

## Figures and Tables

**Table 1:** Characteristics of the study population. Data expressed as correlation coefficient (*p*-value).

	All ^1^	Boys ^2^	Girls ^2^	*p*-Value for Boys vs. Girls
Number (N)	11234	5974	5260	
Age (years)	13.74 (0.02)	13.72 (0.03)	13.76 (0.03)	0.365
Height (cm)	159.82 (0.11)	162.90 (0.99)	156.32 (0.11)	<0.001
Weight (kg)	53.34(0.14)	56.60 (0.15)	49.64 (0.16)	<0.001
WC (cm)	69.48 (0.10)	71.75 (0.12)	66.90 (0.13)	<0.001
BMI	20.59 (0.04)	21.00 (0.05)	20.13 (0.05)	<0.001
WHtR (m/m)	0.435 (0.001)	0.441 (0.001)	0.428 (0.001)	<0.001
High BMI (%)	7.51 (0.27)	7.95 (0.37)	7.23 (0.40)	0.189
High WHtR (%)	11.91 (0.34)	22.93 (0.49)	15.43 (0.52)	<0.001
SBP (mmHg)	106.89 (0.10)	109.12 (0.13)	103.36 (0.14)	<0.001
DBP (mmHg)	66.01 (0.09)	66.41 (0.09)	65.56 (0.12)	<0.001
Hypertension (%)	6.93 (0.22)	7.11 (0.31)	6.59 (0.33)	<0.001
Energy intake (kcal)	2084.28 (8.37)	2305.39 (11.06)	1838.21 (11.67)	<0.001
Protein intake (g)	75.20 (0.40)	84.29 (0.53)	65.09 (0.56)	<0.001
Fat intake (g)	55.62 (0.35)	61.97 (0.47)	48.55 (0.50)	<0.001
Carbohydrate intake (g)	317.32 (1.22)	347.76 (1.62)	283.45 (1.71)	<0.001
Sodium intake (mg)	3650.29 (20.96)	4082.25 (28.06)	3169.53 (29.61)	<0.001
Saturated fatty acid intake (g)	20.00 (0.19)	22.30 (0.26)	17.41 (0.27)	<0.001
Unsaturated fatty acid intake (g)	32.49 (0.30)	36.51 (0.40)	27.99 (0.43)	<0.001
Dietary fiber intake (g)	19.28 (0.15)	21.08 (0.21)	17.27 (0.22)	<0.001

^1^ Adjusted for sex and age. ^2^ Adjusted for age. WC: waist circumference, BMI: body mass index, WHtR: waist-to-height ratio. SBP: systolic BP, DBP: diastolic BP.

**Table 2:** Correlation between body composition and nutrition intake. Data expressed as correlation coefficient (*p* value).

	Height	Weight	WC	BMI	WHtR
Energy (kcal)	0.230	0.163	0.109	0.058	0.005
	(<0.001)	(<0.001)	(<0.001)	(<0.001)	(0.743)
Protein (g)	0.196	0.160	0.122	0.080	0.004
	(<0.001)	(<0.001)	(<0.001)	(<0.001)	(0.848)
Fat (g)	0.192	0.145	0.095	0.063	0.002
	(<0.001)	(<0.001)	(<0.001)	(<0.001)	(0.897)
Carbohydrate (g)	0.191	0.116	0.071	0.071	0.005
	(<0.001)	(<0.001)	(<0.001)	(<0.001)	(0.786)
Sodium (mg)	0.204	0.170	0.126	0.087	0.028
	(<0.001)	(<0.001)	(<0.001)	(<0.001)	(0.032)
Saturated fatty acid (g)	0.126	0.103	0.057	0.041	0.024
	(<0.001)	(<0.001)	(<0.001)	(0.046)	(0.065)
Unsaturated fatty acid (g)	0.045	0.054	0.018	0.004	0.012
	(0.049)	(0.034)	(0.115)	(0.827)	(0.236)
Dietary fiber (g)	0.025	0.005	0.024	0.012	0.025
	(0.072)	(0.735)	(0.112)	(0.245)	(0.081)

Adjusted for sex and age. WC: waist circumference, BMI: body mass index, WHtR: waist-to-height ratio.

**Table 3:** Multiple regression analysis for blood pressure.

Dependent Variable	Independent Variable	Beta	Standard Error	Standardized-Beta	*t*	*p*-Value
SBP	Sex	−2.699	0.193	−0.131	−13.989	<0.001
	Age	−0.175	0.050	−0.044	−3.508	<0.001
	Height	0.208	0.013	0.234	16.060	<0.001
	BMI	0.775	0.060	0.286	12.934	<0.001
	WHtR	2.226	3.817	0.012	0.583	0.560
DBP	Sex	0.151	0.180	0.008	0.839	0.401
	Age	0.633	0.046	0.178	13.613	<0.001
	Height	0.120	0.012	0.152	9.930	<0.001
	BMI	0.313	0.056	0.130	5.609	<0.001
	WHtR	−3.663	3.54	−0.023	−1.031	0.303

SBP: systolic blood pressure, DBP: diastolic pressure, BMI: body mass index, WHtR: waist height ratio.

**Table 4:** Correlation between blood pressure and nutrition intake with adjustments for sex and age. Data expressed as correlation coefficient (*p* value).

Additional Adjustment		Energy Intake	Protein Intake	Fat Intake	Carbohydrate Intake	Sodium Intake	SFA Intake	USFA Intake	DF Intake
None	SBP	0.107	0.102	0.077	0.091	0.096	0.063	0.012	0.029
		(<0.001)	(<0.001)	(<0.001)	(<0.001)	(<0.001)	(<0.001)	(0.401)	(0.188)
	DBP	0.036	0.042	0.029	0.022	0.051	0.032	0.021	0.008
		(0.001)	(<0.001)	(0.010)	(0.044)	(<0.001)	(0.048)	(0.154)	(0.651)
BMI	SBP	0.044	0.038	0.024	0.042	0.032	0.043	0.003	0.011
		(<0.001)	(0.002)	(0.032)	(<0.001)	(0.003)	(0.001)	(0.804)	(0.317)
	DBP	0.021	0.022	0.011	0.013	0.030	0.009	0.010	0.001
		(0.053)	(0.041)	(0.258)	(0.201)	(0.005)	(0.497)	(0.435)	(0.907)
Height	SBP	0.031	0.038	0.010	0.027	0.027	0.018	0.005	0.014
		(0.003)	(0.002)	(0.285)	(0.016)	(0.016)	(0.216)	(0.775)	(0.271)
	DBP	−0.040	−0.022	−0.036	−0.042	−0.015	0.013	0.011	0.006
		(<0.001)	(0.051)	(0.005)	(<0.001)	(0.157)	(0.549)	(0.483)	(0.772)
BMI and	SBP	0.009	0.010	−0.005	0.012	0.001	−0.022	−0.020	0.023
Height		(0.391)	(0.339)	(0.613)	(0.245)	(0.884)	(0.129)	(0.154)	(0.102)
	DBP	−0.026	−0.014	−0.029	−0.025	−0.009	−0.021	−0.019	−0.008
		(0.109)	(0.177)	(0.122)	(0.106)	(0.371)	(0.152)	(0.192)	(0.569)

SFA: saturated fatty acid, USFA: unsaturated fatty acid, DF: dietary fiber. BMI: body mass index, SBP: systolic blood pressure, DBP: diastolic pressure.

**Table 5:** Differences between normotensive and hypertensive groups. Data expressed as mean (standard error).

	Normotension Group	Hypertension Group	*p*-Value
Number (N)	10455	779	
Age (years)	13.74 (0.03)	13.72 (0.10)	0.544
Height (cm)	159.80 (0.09)	160.10 (0.32)	0.360
Weight (kg)	52.98 (0.11)	60.39 (0.44)	<0.001
WC (cm)	69.17 (0.09)	75.15 (0.36)	<0.001
BMI	20.47 (0.04)	22.80 (0.14)	<0.001
WHtR	0.433 (0.001)	0.464 (0.002)	<0.001
High BMI (%)	6.69 (0.28)	19.36 (1.00)	<0.001
High WHtR (%)	12.58 (0.33)	29.00 (1.32)	<0.001
SBP (mmHg)	105.86 (0.09)	123.30 (0.35)	<0.001
DBP (mmHg)	65.26 (0.08)	77.93 (0.32)	<0.001
Energy intake (kcal)	2075.73 (33.54)	2199.58 (9.46)	0.005
Protein intake (g)	75.01 (1.64)	77.81 (0.47)	0.031
Fat intake (g)	55.36 (1.43)	59.15 (0.41)	0.008
Carbohydrate intake (g)	315.94 (4.96)	335.89 (1.41)	0.007
Sodium intake (mg)	3641.06 (85.99)	3774.80 (24.25)	0.029
Saturated fatty acid intake (g)	19.87 (0.82)	21.70 (0.21)	0.044
Unsaturated fatty acid intake (g)	32.42 (1.28)	33.49 (0.33)	0.465
Dietary fiber intake (g)	19.26 (0.16)	19.59 (0.65)	0.723

Adjusted for sex and age. WC: waist circumference, BMI: body mass index, WHtR: waist height ratio, SBP: systolic blood pressure, DBP: diastolic blood pressure.

## Data Availability

Publicly available datasets were analyzed in this study. These data can be found here: (https://www.kdca.go.kr) (accessed on 24 September 2022).
